# Short-term effects of intravitreal anti-vascular endothelial growth factor agents on body weight and multiple systems after treatment for retinopathy of prematurity

**DOI:** 10.3389/fped.2022.1077137

**Published:** 2023-01-25

**Authors:** Jing Chen, Qingfei Hao, Jing Zhang, Yanna Du, Haoming Chen, Xiuyong Cheng

**Affiliations:** Department of Neonatology, The First Affiliated Hospital of Zhengzhou University, Zhengzhou, China

**Keywords:** body weight gain, intravitreal anti-VEGF, multiple systems, retinopathy of prematurity, ranibizumab, aflibercept

## Abstract

**Objectives:**

This study's goal was to assess the short-term effect on body weight and multiple systems following intravitreal injections of ranibizumab and aflibercept for retinopathy of prematurity (ROP).

**Methods:**

We retrospectively assessed infants with ROP who received intravitreal anti-vascular endothelial growth factor agents (VEGF) treatment at our hospital. They were classified into 2 groups based on the drugs administered: the intravitreal ranibizumab (IVR) group and the intravitreal aflibercept (IVA) group. The body weight (BW) gains for the pre-treatment week, the 1st week after treatment, and the 2nd week after treatment were compared for each group. Additionally, other parameters such as blood pressure, heart rate, oxygen concentration, volume of milk and output of urine at four time points were also measured. We used repeated measurement analysis of variance analyzed these data.

**Results:**

In total, 95 preterm infants were recruited, including 51 cases in the IVR group and 44 cases in the IVA group. The BW gain for the 1st week after treatment was significantly lower than the pre-treatment week in each group (*P *< 0.05), while there was no decrease in weekly BW gain in the 2nd week after treatment compared with that pre-treatment week. Based on the comparison between groups, the BW gain in the IVR group was significantly higher than in the IVA group in the second post-treatment week. Repeated measurement analysis of variance showed that there were no significant differences in blood pressure, heart rate, oxygen concentration, volume of milk and output of urine in both groups over time.

**Conclusions:**

IVR and IVA could have a short-term inhibitive effect on body weight gain in infants after treatment for ROP, whereas there is no significant impact on other systems.

## Introduction

Retinopathy of prematurity (ROP), one of the leading causes of childhood blindness, is an abnormal proliferative condition of the immature retinal vessels that occurs in preterm and low birth weight infants (1). Every year, the number of ROP requiring treatment is increasing worldwide. As the gold standard for treating ROP, laser photocoagulation has some side effects, such as anisometropia, visual field impairment and a rise in myopia (2). According to a multicenter randomized clinical trial in 2011, anti-vascular endothelial growth factor agents (VEGF) injection intravitreally was found to be efficient in treating ROP (3), and subsequent reports revealed that anti-VEGF treatment was less likely to cause ocular complications mentioned above, compared to laser photocoagulation (4, 5). In light of these studies, anti-VEGF is emerging as a promising therapeutic option in the management of ROP. However, it is important to note that recent clinical studies suggested that these anti-VEGF drugs can subsequently elude the eye and pass into the systemic circulation following intravitreal injections (6, 7). VEGF is essential for the development of organs like the lung, heart, brain, and kidney, and thus the possible adverse systemic impacts need to be taken into account.

There are several clinical studies have examined the effects of intravitreal anti-VEGF injections for ROP on body weight (BW) gain, but most of these studies focused on the long-term effect (8, 9). Since numerous factors other than the anti-VEGF injection, including meal volume and disease state, may have an impact on long-term BW growth, the direct effect of anti-VEGF injection on body weight gain may be better assessed by examining short-term weight gain. The study by Obata S et al. (10) first reported the short-term inhibitive effects of intravitreal bevacizumab on body weight. And to the best of our knowledge, this is the only research on the short-term BW gain and systemic adverse effects caused by intravitreal anti-VEGF injection for ROP, which needs to be discussed. Therefore, we conducted further investigation based on the Obata S et al.'s study (10).

In this study, we aimed to investigate the short-term effect on body weight and multiple systems after intravitreal injections of ranibizumab and aflibercept for ROP.

## Materials and methods

### Study subjects

This study was a retrospective, single-center cohort study, approved by the Ethics Committee of the First Affiliated Hospital of Zhengzhou University. Informed consent was obtained from the guardians before anti-VEGF treatment was administered. We selected infants with ROP born in our hospital from January 2017 to December 2021 and treated with anti-VEGF therapy. All subjects had a complete medical history and were followed up for at least two weeks after treatment. There were three exclusion criteria: (1) missed weight data on the day of treatment; (2) discharged within two weeks after treatment; (3) suffered from other fundus diseases, chromosomal anomalies, or genetic metabolic diseases.

The definition, diagnosis, and classification related to ROP were referred to international guideline for the screening of retinopathy of prematurity (11).

### Process of treatment

The infants were placed on the operating table with the assistance of an assistant, and oxybuprocaine hydrochloride eye drops were used to administer topical anesthesia. After rinsing of the conjunctival sac, disinfection of the towel, placement of a face opener, and disinfection of the conjunctival sac again with povidone-iodine, ranibizumab (0.025 ml, Novartis Pharma Stein AG, Switzerland), or aflibercept (0.025 ml, Vetter Pharma-Fertigung GmbH & Co. KG, Germany) was injected intravitreally through the pars plana at 1.5 mm posterior to the limbus with a 1 ml syringe. Following injection, tobramycin dexamethasone eye ointment and sterile gauze bandages were applied, and tobramycin eye drops were used postoperatively to prevent infection.

### Data collection

We investigated the factors regarding infants retrospectively from the medical records, including: the basic information (gestational age, birth weight, sex, multiple births, Apgar scores at 1 and 5 min); comorbidities (sepsis, respiratory distress syndrome, neonatal necrotizing enterocolitis, patent ductus arteriosus, bronchopulmonary dysplasia, intracranial hemorrhage); preoperative and postoperative ROP screening results; pulse rate, blood pressure, inhaled oxygen concentration, milk volume, and urine volume at four time points (7 days before treatment, day of treatment, 7 days after treatment, 14 days after treatment). The BW gains for the pre-treatment week (7 days before treatment), the 1st week after treatment (first 7 days following treatment), and the 2nd week after treatment (7 to 14 days following treatment) were also collected.

### Statistical analysis

GraphPad Prism 8.0 software was applied for statistical analyses. The Student's *t*-test was used to compare between groups for the continuous variables with normal distribution, which were provided as mean and standard deviation. Mann-Whitney *U* test was used to compare non-parametric data presented as medians and interquartile ranges. As for the categorical data, the *χ*^2^ test or Fisher's exact test was used to compare the two groups based on the frequencies and percentages. Repeated-measures data were analyzed by repeated-measures analysis of variance. Statistical significance was determined by *P*-values less than 0.05.

## Results

A total of ninety-five infants with ROP treated with anti-VEGF were included, fifty-one of whom were treated with ranibizumab and forty-four with aflibercept. They were classified into the intravitreal ranibizumab (IVR) group and the intravitreal aflibercept (IVA) group. In the IVR group, the mean gestational age was (28.5 ± 1.5) weeks, the mean birth weight was (1071.8 ± 241.2) g, and the corrected gestational age at the time of treatment was (36.4 ± 2.0) weeks. And in the aflibercept group, the mean gestational age was (28.0 ± 1.6) weeks, the mean birth weight was (990.9 ± 217.9) g, and the corrected gestational age at the time of treatment was (36.8 ± 2.3) weeks. The baseline characteristics of the two groups were compared, and the results did not show a statistically significant difference (Table 1).

**Table 1:** Comparison of baseline characteristics between the IVR group and IVA group.

	IVR group (*n* = 51)	IVA group (*n* = 44)	*t/χ*^2^*/Z*值	*P*-value
Gestational age, week, mean (SD)	28.5 ± 1.5	28.0 ± 1.6	1.601	0.113
Birth weight, g, mean (SD)	1071.8 ± 241.2	990.9 ± 217.9	1.703	0.092
Sex (male/female)	31/20	27/17	0.003	0.954
Multiple birth, *n* (%)	15 (29.4)	10 (22.7)	0.544	0.461
Apgar score at 1 min, median (IQR)	7.0 (5.0, 8.0)	7.0 (4.3,8.0)	−0.915	0.360
Apgar score at 5 min, median (IQR)	8.0 (8.0,9.0)	8.0 (6.0,9.0)	−0.953	0.341
Respiratory distress syndrome, *n* (%)	50 (98.0)	44 (100)	0.872	0.350
Sepsis, *n* (%)	43 (84.3)	31 (70.5)	2.635	0.105
Necrotizing enterocolitis, *n* (%)	9 (17.6)	10 (22.7)	0.381	0.537
Patent ductus arteriosus, *n* (%)	16 (31.4)	15 (34.1)	0.079	0.778
Bronchopulmonary dysplasia, *n* (%)	35 (68.6)	33 (75)	0.472	0.492
Intraventricular hemorrhage, *n* (%)	46 (90.2)	35 (79.5)	2.132	0.144
Postmenstrual age at treatment, week, mean (SD)	36.4 ± 2.0	36.8 ± 2.3	−1.049	0.297
Birth to treatment period, days, mean (SD)	55.4 ± 15.7	61.9 ± 17.7	−1.911	0.059
Zone at treatment (I/II)	31/20	19/25	2.936	0.087

IVR, intravitreal ranibizumab; IVA, intravitreal aflibercept.

**Figure 1:**
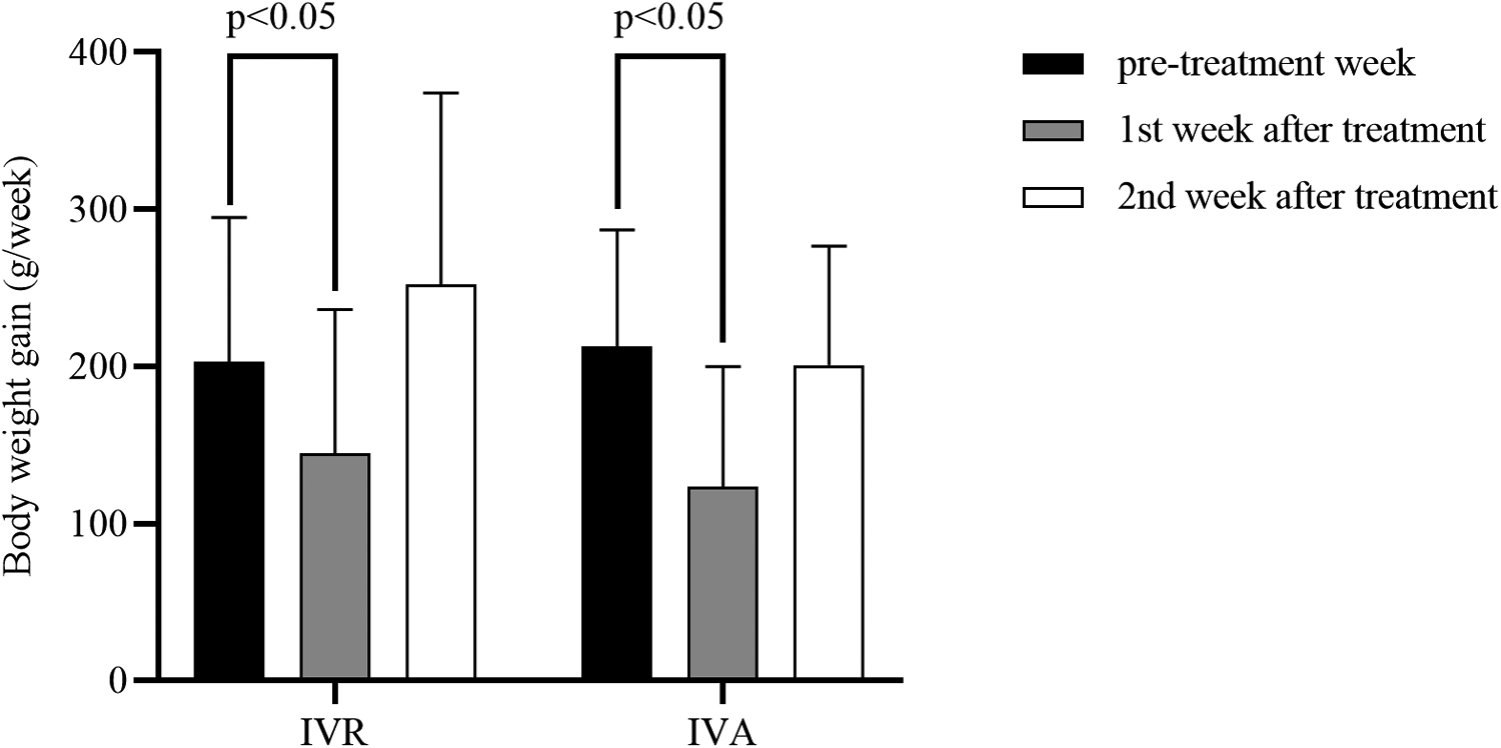
Comparison of weekly BW gain before and after treatment in the two groups.

**Figure 2:**
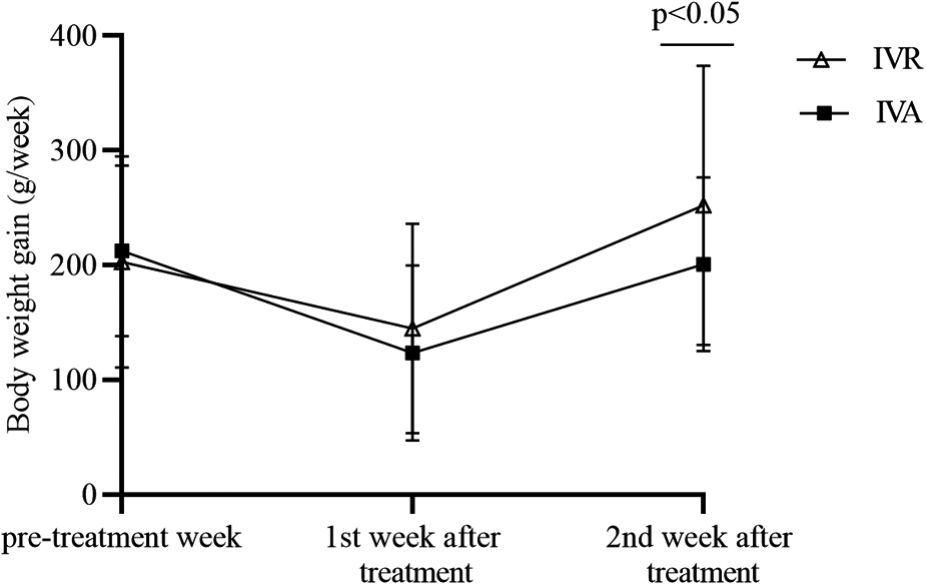
Comparison of weekly BW gain between the two groups.

Table 2 and Figure 1 displayed the body weight gains for the pre-treatment week, the first post-treatment week, and the 7 to 14 days period following treatment. We used the repeated-measures analysis of variance to determine the effect of different intervention measures on the BW gain of the infants over time. The results revealed that there was a statistically significant interaction between group and time (*F* = 4.474, *P* = 0.014), and then time and intervention factors were tested separately. In the pre-treatment week and the first post-treatment week, BW gain did not differ significantly between the two groups (*P* > 0.05). The BW gain in the IVR group was significantly higher than in the IVA group in the 2nd week after treatment (Figure 2). In addition, a statistically significant effect of time was found (*F* = 36.797, *P *< 0.001). Further comparisons at the pairwise level showed that there was a significant decrease in weekly BW gain during the first week after treatment compared with the pre-treatment week in both groups, while there was no reduction in weekly BW gain in the 7 to 14 days after treatment compared with that pre-treatment week.

**Table 2:** Comparison of weekly BW gain before and after treatment in the two groups.

	IVR (*n* = 51)	IVA (*n* = 44)
Pre-treatment week, g/week, mean (SD)	202.9 ± 92.1	212.6 ± 74.3
1st week after treatment, g/week, mean (SD)	144.7 ± 91.6	123.4 ± 76.6
2nd week after treatment, g/week, mean (SD)	252.3 ± 121.6	200.8 ± 75.8

BW, body weight; IVR, intravitreal ranibizumab; IVA, intravitreal aflibercept.

**Figure 3:**
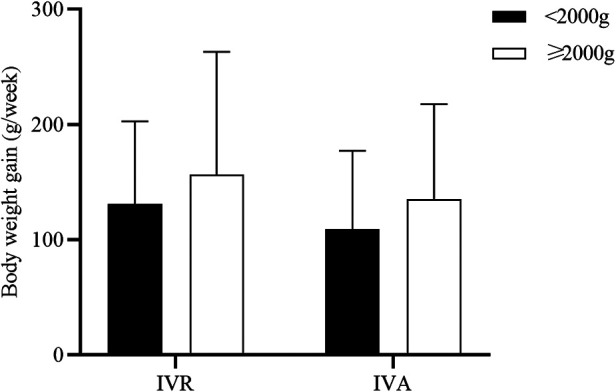
Comparison of the weekly BW gains for the first post-treatment week based on the BW at treatment.

In addition, we compared with infants whose BW at treatment < 2000 g and those whose BW ≥ 2000 g in order to identify whether anti-VEGF affected BW gain differently based on the BW at treatment (Figure 3). The results indicated that there were no statistically significant in the weekly BW gains for the 1st week after treatment between infants with BW < 2000 g at treatment and infants with BW ≥ 2000 g in the IVR group (131.1 g vs. 156.8 g) and in the IVA group (109.1 g vs. 135.3 g).

**Figure 4:**
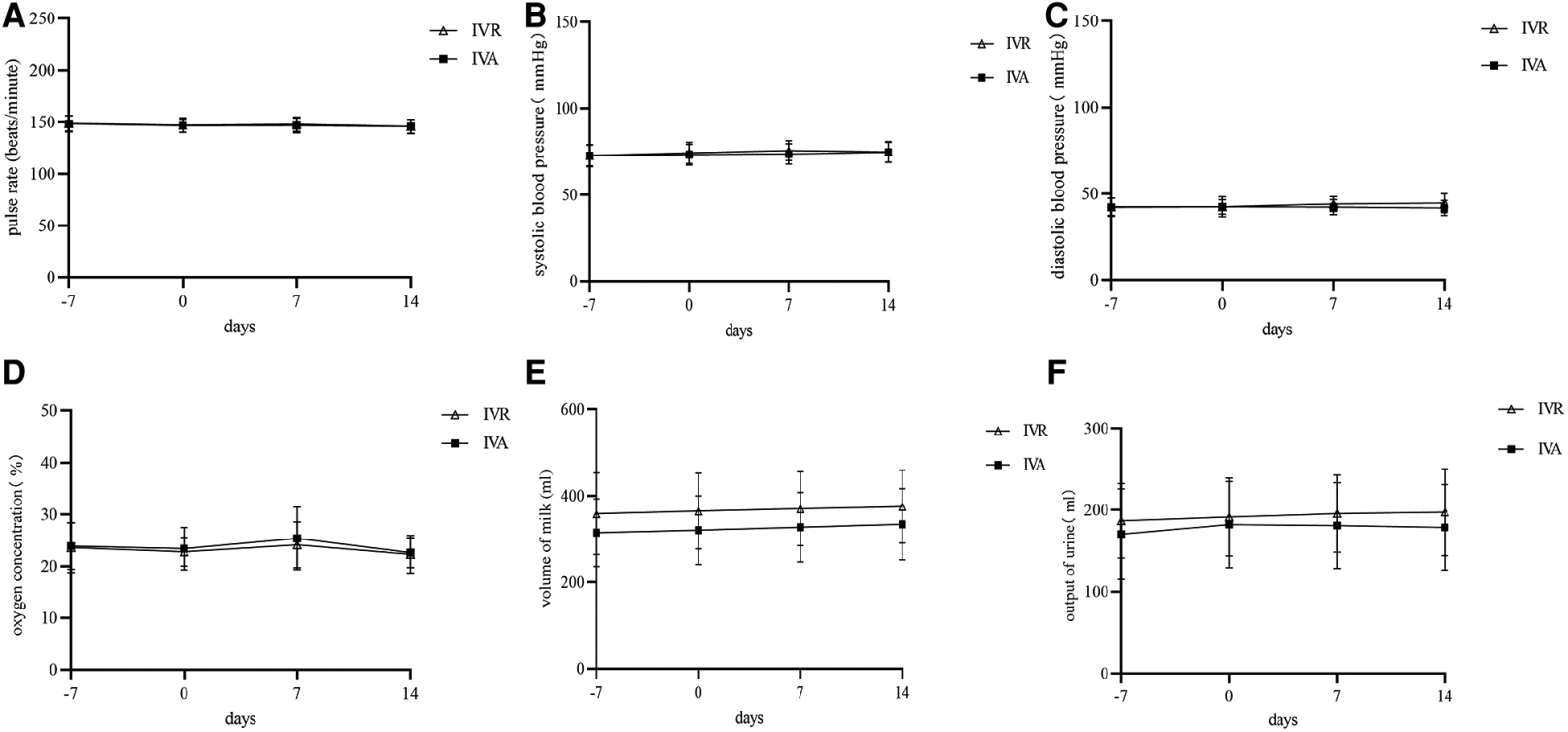
Comparison of parameters other than BW gain before and after treatment in the two groups. **A-F**, Changes in pulse rate (**A**), systolic blood pressure (**B**), diastolic blood pressure (**C**), oxygen concentration (**D**), volume of milk (**E**), and output of urine (**F**).

To explore the effects of intravitreal anti-VEGF injections on multiple systems, we compared the changes in pulse rate, blood pressure, inhaled oxygen concentration, volume of milk and output of urine at four time points (Figure 4). There were no statistically significant differences in these factors in both groups over time.

## Discussion

As far as we know, this is the first clinical report to investigate the short-term influence of IVR and IVA on the BW gain and multiple systems in infants with ROP based on the Obata S et al.'s study of the effect of intravitreal bevacizumab (10). In this single-center retrospective study, our results suggested that BW gain in infants with ROP was inhibited for a short term after IVR and IVA, whereas the parameters of other systems were not influenced.

The effectiveness and security of intravitreal anti-VEGF injection for the treatment of ROP have been well established in numerous studies (12, 13). And in the present study, all infants also showed improvement in fundus anatomical outcomes after anti-VEGF treatment. The use of anti-VEGF drugs is becoming a first-line treatment option for ROP. However, several toxic side effects have been reported with these new agents. In recent years, there has been growing concern that anti-VEGF drugs could make their way into the circulatory system through the blood-eye barrier. The RAINBOW trial demonstrated for the first time that ranibizumab could be detected in serum after intravitreal injection (14). Similarly, several subsequent studies have indicated that other anti-VEGF also could be detected in the blood, as well as a decrease in VEGF concentration and insulin-like growth factor-1 (IGF-1) levels in serum after intravitreal injection (15–17). Because VEGF plays an important role in the early neonatal period growth, anti-VEGF treatment can alter growth parameters such as head circumference, weight and body length by slowing down the growth rate. Previous clinical studies have reported that anti-VEGF can prevent BW gain in infants for months or even years after intravitreal injection for ROP (8, 9). Since numerous factors other than the anti-VEGF injection, including meal volume, social factors, and disease state, may have an impact on long-term BW growth, the direct effect of anti-VEGF injection on body weight gain may be better assessed by examining short-term weight gain. Obata S et al. reported that intravitreal bevacizumab injection had an inhibitory effect on short-term weight gain (10). In our study population, IVR and IVA both inhibited the BW gain in the 1st week after treatment. We suppose that the slower weight gain was likely due to a systemic side effect of IVR and IVA, because the differences in milk and urine volume were not statistically significant, thus excluding the effect of feeding volume and urine volume on weight. Also, Obata S et al. (10) found that infants with low BW at treatment were more likely to experience the inhibitory effect of IVB on BW gain, while our study didn't discover similar trend. This may be due to the higher mean weight at the treatment of our study subjects.

There are differences in the pharmacokinetics of different drugs. Compared to ranibizumab, aflibercept has a longer half-life and a higher molecular mass. A study by Furuncuoglu U et al. confirmed aflibercept was still detectable in the blood circulation at 4 weeks after intravitreal injection for ROP (15). We speculated might aflibercept cause greater delays in growth and development in preterm infants, because the ranibizumab was cleared from the circulation more rapidly.

According to the results of this study, both IVA and IVR inhibited weight gain in ROP infants for one week after treatment. While there was no decrease in weekly BW gain in the 2nd week after treatment compared with that pre-treatment week, the IVR group had a significantly higher BW gain than the IVA group during the 7 to 14 days after treatment, which may suggest aflibercept resulted in greater suppression of BW gain.

In addition to the effects on growth parameters, an animal study reported that anti-VEGF influenced organ development by increasing the specific expression of VEGF mRNA in the lung, kidney, heart, and brain (18). Le Cras et al. indicated that inhibition of the VEGF signaling pathway caused pulmonary hypertension and BPD in neonatal rats by reducing pulmonary vascular growth and impairing postnatal alveolarization (19). Furthermore, a clinical study demonstrated that IVB might increase the need for ventilatory support in infants with BPD (20).

Also, there has been great concern about the cardiovascular toxicities. The mean arterial blood pressure in rats has also been shown to decrease following intravenous administration of VEGF. There are also reports of cardiovascular side effects that are associated with intravitreal anti-VEGF therapy, such as arterial thromboembolism, systemic hypertension and congestive heart failure (21–23).

Regarding the effect of anti-VEGF on neurological development, some studies suggested that IVB could cause early neurodevelopmental disorders (24, 25). While Stahl A et al. followed sixteen ROP infants treated with IVR for two years and found no relationship between IVR and neurodevelopmental disabilities (26). Therefore, a better understanding of the role of anti-VEGF in neurodevelopmental disorders requires further analysis.

Recently, animal studies have shown that anti-VEGF causes loss of glomerular endothelial cells and alterations in glomerular filtration rate (18). Although kidney damage evidenced by microangiopathy and proteinuria has been reported in adult human patients receiving anti-VEGF for cancer treatment (27), there is no research demonstrating renal damage in infants with ROP following intravitreal anti-VEGF injection treatment.

In the present study, a comparison of pulse rate, blood pressure, oxygen concentration, volume of milk, and urine output before and after treatment was conducted. Neither group had statistically significant differences in these factors over time. The oxygen concentrations of the two groups increased in the first post-treatment week without statistical significance, which was consistent with the result of Obata S et al.'s study on IVB (10). This indicated that anti-VEGF quite likely cause an adverse respiratory effect in ROP neonates. In order to investigate the impacts of anti-VEGF on neonatal lung development, further large sample studies are needed.

There are limitations to our study because of the small sample size and the retrospective, single-center design. Another limitation is that we did not assess changes in other growth parameters such as head circumference and body length. More large-sample and multicenter prospective studies are needed to confirm the systemic safety of intravitreal injections of anti-VEGF.

In conclusion, IVR and IVA could have a short-term inhibitive effect on body weight gain in infants with ROP, whereas there is no significant impact on other systems. The systemic safety of anti-VEGF agents in infants must be clearly defined in adequately powered, well-designed clinical trials.

## Data Availability

The original contributions presented in the study are included in the article/Supplementary Material, further inquiries can be directed to the corresponding author/s.
